# Metagenomic characterization of the effect of feed additives on the gut microbiome and antibiotic resistome of feedlot cattle

**DOI:** 10.1038/s41598-017-12481-6

**Published:** 2017-09-25

**Authors:** Milton Thomas, Megan Webb, Sudeep Ghimire, Amanda Blair, Kenneth Olson, Gavin John Fenske, Alex Thomas Fonder, Jane Christopher-Hennings, Derek Brake, Joy Scaria

**Affiliations:** 1 Department of Veterinary and Biomedical Sciences, South Dakota State University, Brookings, South Dakota USA; 2 South Dakota Center for Biologics Research and Commercialization, Brookings, South Dakota USA; 3 Department of Animal Science, South Dakota State University, Brookings, South Dakota USA

## Abstract

In North America, antibiotic feed additives such as monensin and tylosin are added to the finishing diets of feedlot cattle to counter the ill-effects of feeding diets with rapidly digestible carbohydrates. While these feed additives have been proven to improve feed efficiency and reduce liver abscess incidence, how these products impact the gastrointestinal microbiota is not completely understood. In this study, we analyzed the impact of providing antibiotic feed additives to feedlot cattle using metagenome sequencing of treated and control animals. Our results indicate that use of antibiotic feed additives does not produce discernable changes at the phylum level. However, treated cattle had reduced abundance of gram-positive bacteria at the genus level. The abundance of Ruminococcus, Erysipelotrichaceae and Lachnospiraceae in the gut of treated steers was reduced. Functional analysis of the data indicates that there was only minimal impact due to the treatment in the rumen. Genes involved in detoxification were significantly increased in the rumen of AB steers. But the relative abundance of these genes was < 0.3%. However, our results did not show any correlation between the presence of antimicrobial resistance genes in the gut microbiota and the administration of antibiotic feed additives.

## Introduction

Commensal microbes inhabiting the mammalian gut are associated with immune development^1^, nutrient supply^2^, prevention of pathogenic diseases^3^, and overall health of the host. The nutritional dependency on microbial population is particularly important in the case of ruminants as the end products of microbial fermentation in the rumen provide most of the energy required by the animal. Alternatively, dietary practices influence the structure of the microbial community^4,5^. Cattle in feedlots are generally fed a finishing ration that contains up to 90% rapidly digestible carbohydrates. This elevates incidences of acidosis^6^ and liver abscesses^7^, which have a detrimental effect on animal health and beef production. In North America, antibiotic feed additives such as monensin and tylosin are added to the finishing diets to counter the ill-effects of feeding a carbohydrate-rich diet^8–11^. Monensin is a polyether antibiotic isolated from *Streptomyces cinnamonensis* ^12^. Tylosin is a macrolide antibiotic and bacteriostatic feed additive^13^. While these additives are proven to improve feed efficiency, reduce liver abscess incidence, and control coccidiosis, the understanding of how these additives influence the microbial dynamics of the rumen is incomplete. Use of antibiotic feed additives in livestock production is a controversial topic due to the concerns that use of antibiotic feed additives could expand the antibiotic resistome in the animal’s gastrointestinal tract (GIT). The antibiotic resistome is the collection of antibiotic resistance genes and their precursors in both pathogenic and non-pathogenic bacteria in a given environment^14^.

The alterations to the gut microbes in ruminants induced by energy-rich diets and antibiotic feed additives have been studied previously^6,15–19^. However, the methodologies used in these studies were anaerobic culture, terminal restriction fragment length polymorphism (TRFLP), and 16S rRNA-based polymerase chain reaction (PCR). These techniques lack the ability to analyze whole microbial populations and could only provide information that is limited to few marker organisms. With the recent advances in next-generation sequencing techniques, 16S rRNA gene amplicon sequencing is used to map out the microbial communities in the ruminant gut^20–25^. However, classifying the microbial community based on a single gene could be challenging and often the resolution is compromised especially when the species are closely related. This method is also inherently limited because of the bias induced by PCR while amplifying the gene. Furthermore, the predicted functional characteristics of the microbial communities using 16S rRNA gene amplicon sequencing might have low accuracy. Shotgun metagenomics sequencing, albeit expensive, is a reliable alternative tool that could provide a comprehensive and high-resolution analysis of the microbiome and resistome.

This study provides a snapshot of the structure and functional characteristics of the GIT microbiome and resistome of steers that are adapted to a rapidly fermentable carbohydrate-rich diet and were finished with or without antibiotic feed additives. To our knowledge, this is the first report that uses shotgun metagenomic sequencing for studying the microbiome and resistome alterations in the GIT of steers. Importantly, the findings of this study are relevant for beef cattle operations and public health since the management and diet of the steers in this experiment resemble the practices followed in the cattle feeding industry in North America.

## Results

In this study, shotgun metagenomics sequencing of the GIT microbiome of antibiotic feed additive treated and control feedlot cattle was utilized to determine the influence of these feed technologies on the microbiome and antibiotic resistome. The beneficial effect of feeding antibiotics on improving the health and productivity of feedlot cattle on high-grain diets has been well established. However, it is also a controversial practice that could lead to the emergence of antibiotic-resistant bacterial strains. With the advancement in next generation sequencing technologies, the microbial diversity and antibiotic resistome in the GIT of livestock animals could be studied in greater depth. Previous research conducted on the bovine microbiome made use of a 16S sequencing approach primarily to study the bacterial communities in the GIT. Though it was possible to deduct these results at the operational taxonomic units (OTU), shotgun metagenomics provides better resolution of the taxonomical data to the genera level and even to species level. However, the disadvantage of next generation based metagenome sequencing is that platforms such as Hiseq would have to be used to account for the presence of host genomic DNA contamination. To circumvent this problem, a microbial DNA enrichment method^26^ was used to deplete the host genomic DNA. Using this method, 49 ± 12%, 53 ± 12%, and 15 ± 4% of total DNA could be recovered from the rumen, colon, and cecum samples respectively. This depletion process allowed for metagenomics sequencing using 2 × 250 paired chemistry in Miseq platform, which provides a 100 base longer read than the maximum length available in the HiSeq platform.

### Diversity and richness estimates

Richness refers to the number of species in a microbial community while diversity relates to the number of species and evenness of distribution of the microbial species. The diversity and richness of the microbial population in the rumen, colon, and cecum samples was obtained from five control steers (NA; not provided any antibiotics or growth promoting treatments) and five steers that were provided antibiotic feed additives, growth promoting implants and a beta-agonist (AB) during a research field study. Implants were administered during three production phases; pre-weaning (36 mg zeranol; Ralgro^®^, Merck Animal Health, Madison, NJ), backgrounding (80 mg trenbolone acetate and 16 mg estradiol; Revalor^**®**^-IS, Merck Animal Health), and finishing (200 mg trenbolone acetate and 20 mg estradiol; Revalor^**®**^−200, Merck Animal Health) to the AB steers. Additionally, AB steers received a beta-agonist (Optaflexx^®^-45, Elanco Animal Health, Greenfield, IN) during the final 31 days prior to harvest. Biodiversity of the human gut is reduced by both short-term and long-term use of antibiotics^27,28^. The AB steers were fed antibiotic feed additives, monensin and tylosin, which are known to cause changes in the bacterial population in the animal gut^29,30^. In the present study, we used shotgun metagenomics to study the alterations in the microbial community structure in the GIT of feedlot steers that were fed monensin and tylosin.

### α diversity

Microbial diversity within a local community (α diversity) is measured by either the richness (Chao1) or by diversity indices such as ShannonH or inverse Simpson. The individual sample richness and diversity of rumen, cecum, and colon samples were measured using Chao1, ShannonH, and Inverse Simpson indices by bootstrapping random sequences from the sequences in each sample for 50 times (Table 1). The higher values for ShannonH and inverse Simpson indices indicate that rumen samples from NA steers had a more diversified microbial community. Also, rumen samples from NA steers exhibited higher species richness, as measured by the Chao1 index, than those from AB steers. This suggested that antibiotic treatment might have reduced both community diversity and richness of the microbial population in the rumen. Interestingly, species richness was higher in the colon of AB steers. However, there was no difference observed for richness of the microbial population in the cecum between the treatment groups. Communities had similar diversity in the cecum and colon for both control and treatment groups.

**Table 1:** Alpha-diversity of the microbiome in the GI tract of feedlot cattle.

Steers	Rumen			Colon			Cecum		
	Chao1	ShannonH	Simpson	Chao1	ShannonH	Simpson	Chao1	ShannonH	Simpson
NA Mean	512.2	4.69	0.81	394.0	4.97	0.88	461.6	5.33	0.90
NA SD	31.8	0.70	0.06	76.5	0.31	0.02	52.52	0.48	0.03
AB Mean	463.8	4.28	0.77	479.4	5.03	0.88	432.4	5.16	0.88
AB SD	35.1	0.60	0.05	26.3	0.28	0.02	62.99	0.45	0.03
p value	0.01	0.02	0.01	0.03	0.62	0.69	0.48	0.55	0.31

Samples were collected from rumen, colon, and cecum of the control (NA) and treated (AB) steers. Control steers had a more diversified microbial population in the rumen.

### β diversity

**Figure 1:**
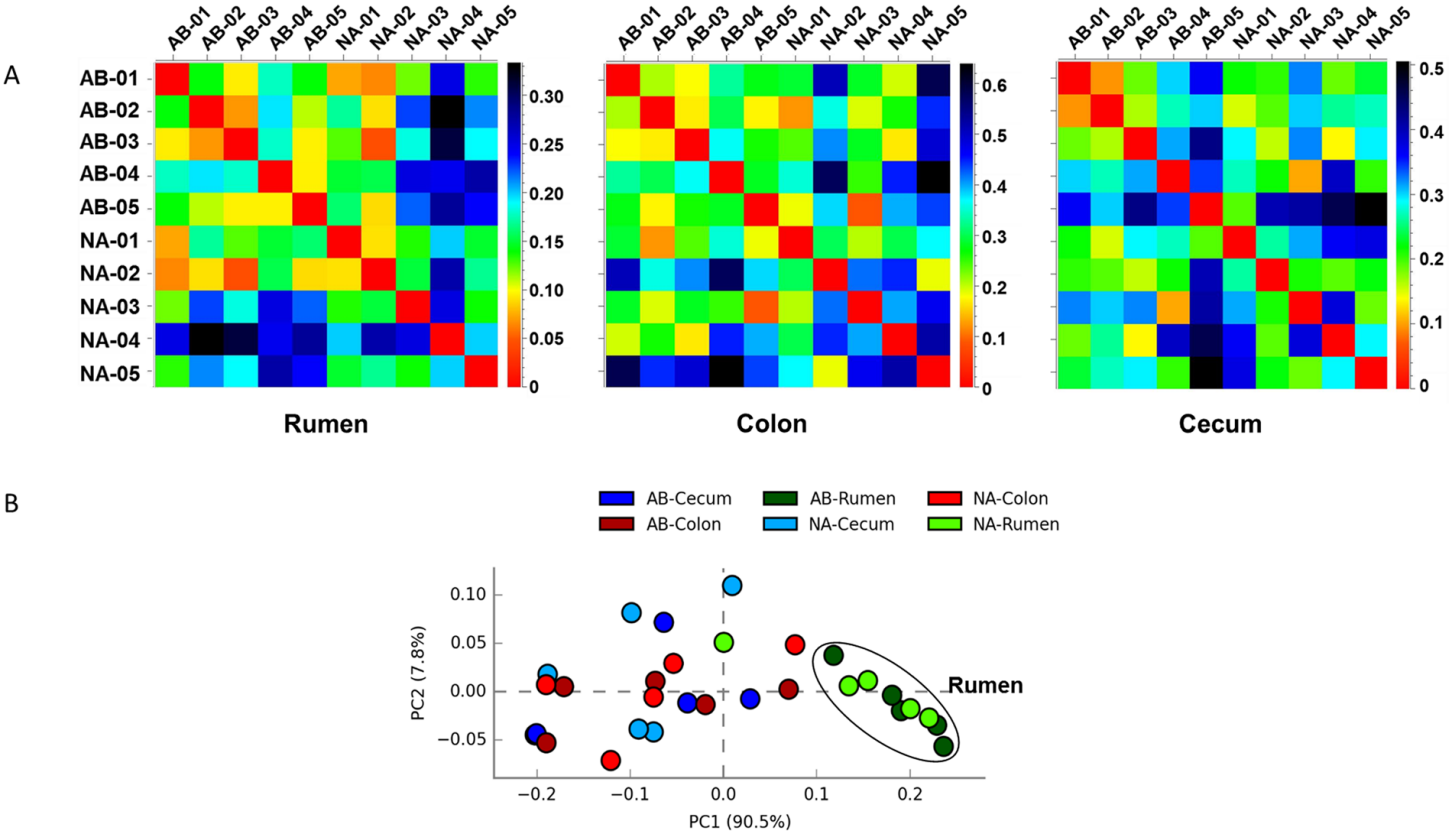
*Microbiome diversity of bacterial population in rumen, colon, and cecum samples obtained from control (NA) and treated (AB) steers.* (**A**) The beta-diversity of the bacterial community was measured using Bray-Curtis index. The X and Y axes denotes the samples. The Bray-Curtis index varies between 0 and 1, where 0 means no difference in species between samples and 1 means all the species are different between samples. (**B**) Principal coordinate analysis (PCA) of the microbial diversity in GI tract of steers showed that the rumen microbiome samples, except for NA-04, clustered together and separated from cecum and colon samples.

Beta diversity (β diversity) is the measurement of diversity between two or more local assemblages; higher the β diversity, larger will be the difference in species identities between the communities. The β diversity among samples was calculated using Bray-Curtis index which measures the dissimilarity between the samples (Fig. 1A). Although a consistent pattern is lacking for between-subject variation, few of the NA steers (NA-03, NA-04, NA-05 in the rumen and NA-02, NA-05 in the colon) exhibited slightly higher diversity when compared to other animals. Additionally, the principal component analysis (PCA) revealed that the all rumen samples from control and treated steers, except for NA-04, clustered together and were separated from cecum and colon samples (Fig. 1B).

### Composition of the rumen, cecum, and colon microbiota at the phylum level

**Figure 2:**
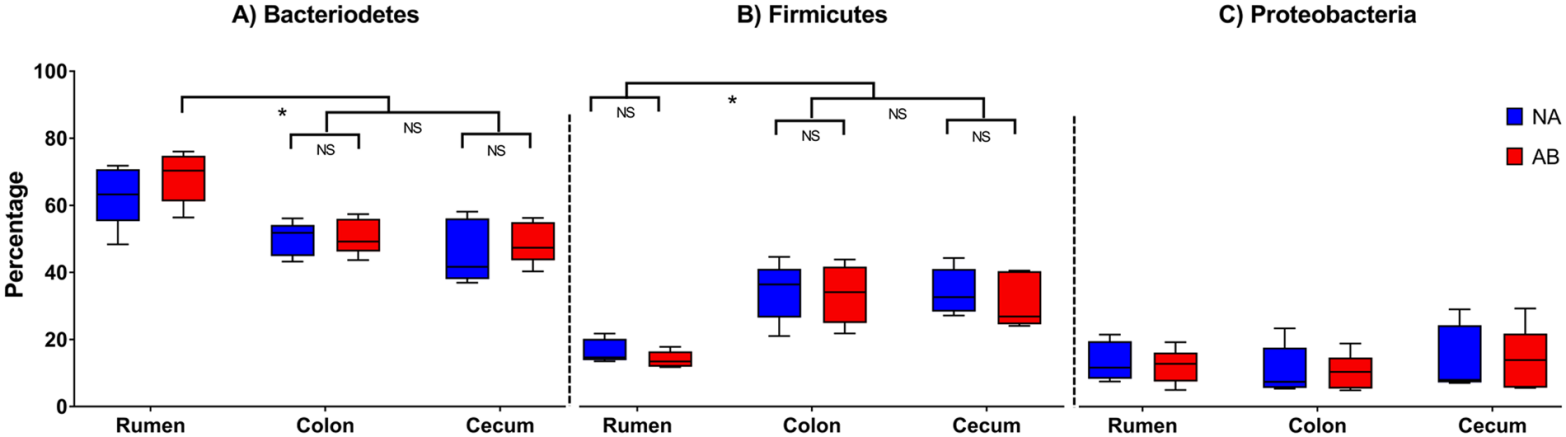
*The phylum-level microbiome composition in the GI tract of steers*. The treated (AB) steers received tylosin and monensin as feed additives while the control (NA) group received no antibiotic feed additives. There was no difference between the two groups within the GIT compartments for the three major phyla; (**A**) Bacteroidetes, (**B**) Firmicutes, and (**C**) Proteobacteria. However, the percentage of Bacteroidetes and Firmicutes varied between the GIT compartments. The whiskers represent 10–90% confidence intervals and the solid black line represents the mean for the samples. NS denotes not significant; *denotes P < 0.05.

Samples from the rumen, colon, and cecum collected from NA and AB steers were analyzed for the phylum-level distribution of the bacterial population. In general, treatments had minimal effect on the distribution pattern of bacteria at the phylum level. However, the bacterial composition altered significantly between the locations in the GIT irrespective of the treatments. The percentage distribution of three major phyla - Bacteroidetes, Firmicutes, and Proteobacteria – are given in Fig. 2. The most abundant phylum in rumen, cecum, and colon was Bacteroidetes. The abundance of Bacteroidetes in the rumen of AB steers was similar to that in the rumen of NA steers but significantly higher (P < 0.05) compared to that in other compartments of both NA and AB groups. The abundance of Firmicutes in the rumen of both NA and AB steers was lower (P < 0.05) than that in cecum and colon for both treatment groups. Although there was no significant difference in the Bacteroidetes and Firmicutes distribution in the rumen of AB steers compared to that of NA steers, the Firmicutes to Bacteroidetes ratio was 0.27 ± 0.10 in NA steers and 0.21 ± 0.06 in AB steers (P < 0.05). There was no difference in the distribution of Proteobacteria between the treatment groups or between the three compartments of GIT.

### Microbiome composition of the rumen, cecum, and colon at the genus level

The shotgun metagenomics sequencing approach provides the potential for analyzing the bacterial community at a higher resolution. Even though the phylum-level distribution did not indicate major shifts in the bacterial community, in-depth analysis at genera-level suggested that treatment with antibiotic feed additives altered the microbial profile in the GIT of steers.

**Figure 3:**
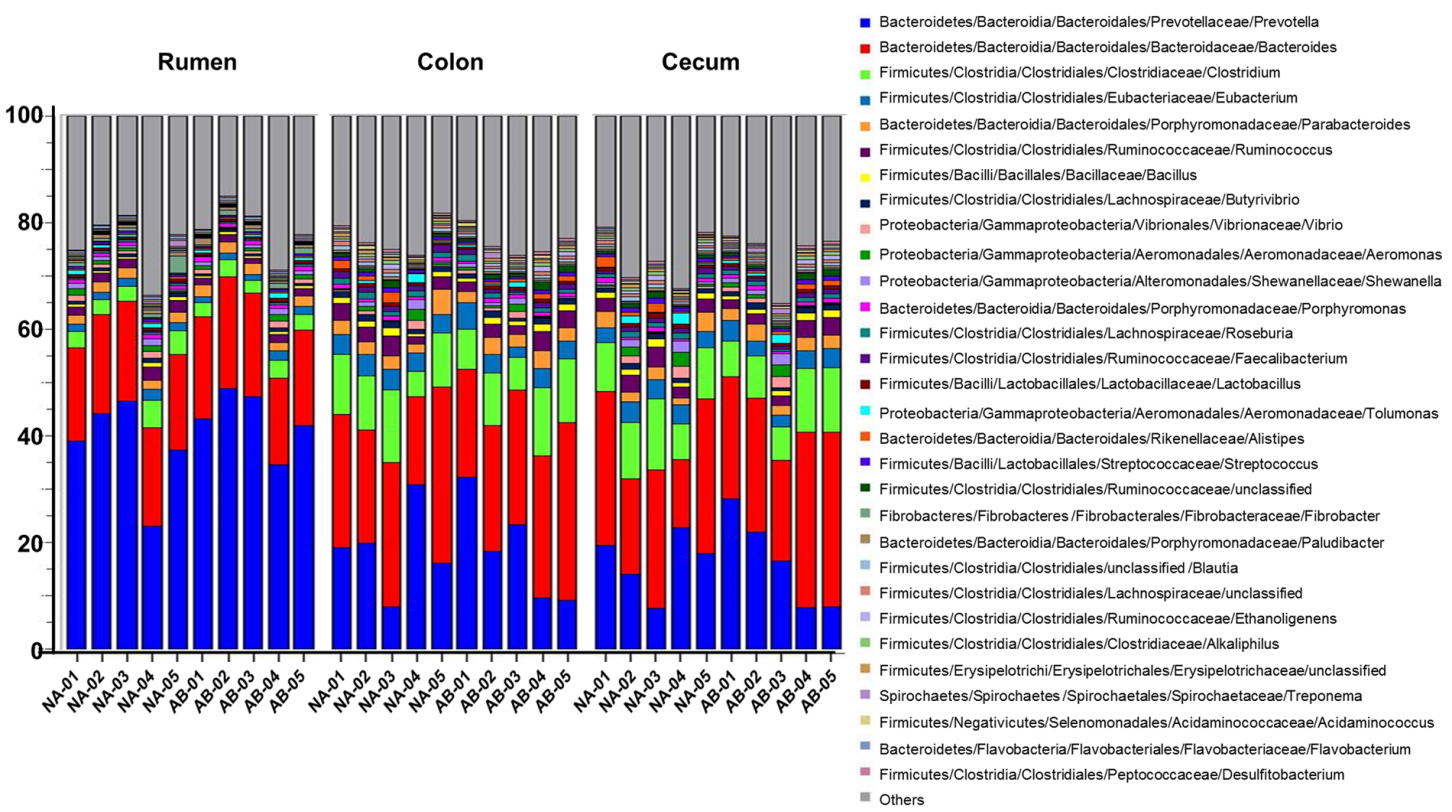
*Genus-level distribution of the microbiome in GI tract of the steers*. Phylogenetic distribution was generated in MG-RAST using RefSeq database with maximum e-value at 10^−5^ value, minimum percentage identity at 60%, minimum alignment length of 30 amino acids and abundance 100. Each color represents one genus. In rumen samples, Prevotella was the most abundant genus, whereas, in colon and cecum, Bacteroides dominated.

Distribution of most abundant genera in rumen, colon, and cecum are given in Fig. 3. There were notable differences in the genus-level distribution between the GI tract compartments. Prevotella was the most abundant genus (39.82%) in the rumen while Bacteroides dominated in the cecum (23.89%) and colon (24.66%). Bacteroides comprised only 18.46% of total population in the rumen. Genus Fibrobacter was markedly abundant in the rumen and contributed to 1.01% of rumen bacteria. In colon and cecum, Fibrobacter made up only 0.18% and 0.19% of the total population respectively. The other abundant genera in the rumen, but present at a lower concentration in cecum and colon, were Paludibacter, Flavobacterium, Mitsuokela, Treponema, Pseudomonas, Selenomonas, and Bifidobacterium.

**Figure 4:**
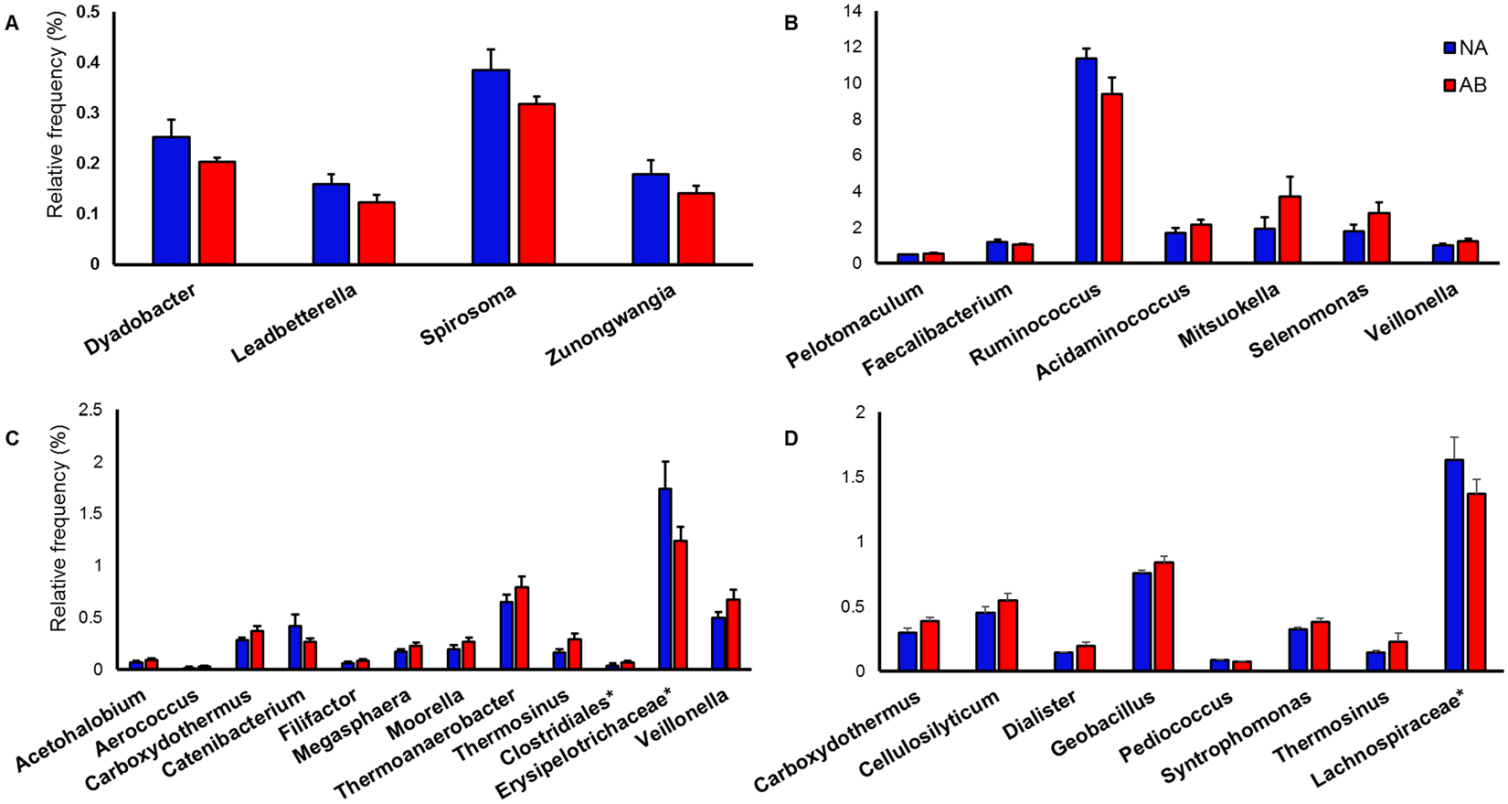
*Alterations in abundance of the bacterial genera in the GI tract of feedlot steers following treatment*. The statistical difference in genera distribution between control (NA) and treated (AB) steers was analyzed using STAMP software. Only those genera that are statistically different (p < 0.05) in abundance are given above. (**A**) genera under Bacteroidetes in the rumen (**B**) genera under Firmicutes in the rumen (**C**) genera under Firmicutes in the colon and (**D**) genera under Firmicutes in the cecum. The genera under Bacteroidetes were similar in both groups of animals in cecum and colon. *Represents unclassified reads derived from families.

To gain further insights, the statistical differences in genera that belong to Bacteroidetes and Firmicutes between NA and AB groups were analyzed using STAMP software. In the rumen, four genera belonging to Bacteroidetes – Spirosoma, Dyadobacter, Leadbetterella, and Zunongwangia – were significantly more abundant in the NA steers (Fig. 4A). On the contrary, there was no difference in the distribution of Bacteroidetes in cecum and colon between treatment groups. Among the Firmicutes, few genera showed a significant difference between treatment groups in the rumen, colon, and cecum (Fig. 4B,C, and D). In the rumen, gram-positive Firmicutes such as Ruminococcus and Fecalibacterium were reduced in the AB steers and were partially replaced by gram-negative members of Negativicutes. These changes were not surprising due to the selective action of monensin and tylosin against gram-positive bacteria. In the colon, gram positive Catenibacterium and unclassified Erysipelotrichacea and in the cecum, unclassified Lachnospiraceae were reduced significantly in AB steers. Similar to the rumen, members belonging to gram-negative Negativicutes increased in abundance in cecum and colon. Interestingly, gram-positive thermophilic organisms belonging to Thermoanaerobacteraceae family also increased in cecum and colon of AB steers. Additionally, in the cecum of AB steers, the abundance of gram-negative genera Cellulosilyticum and Syntrophomonas and thermophilic gram-positive genus Geobacillus also increased. The reason for the resistance pattern exhibited by gram-positive thermophilic organisms to tylosin and monensin is currently unknown.

### Functional analysis of microbiome changes in the GI tract of feedlot cattle

**Figure 5:**
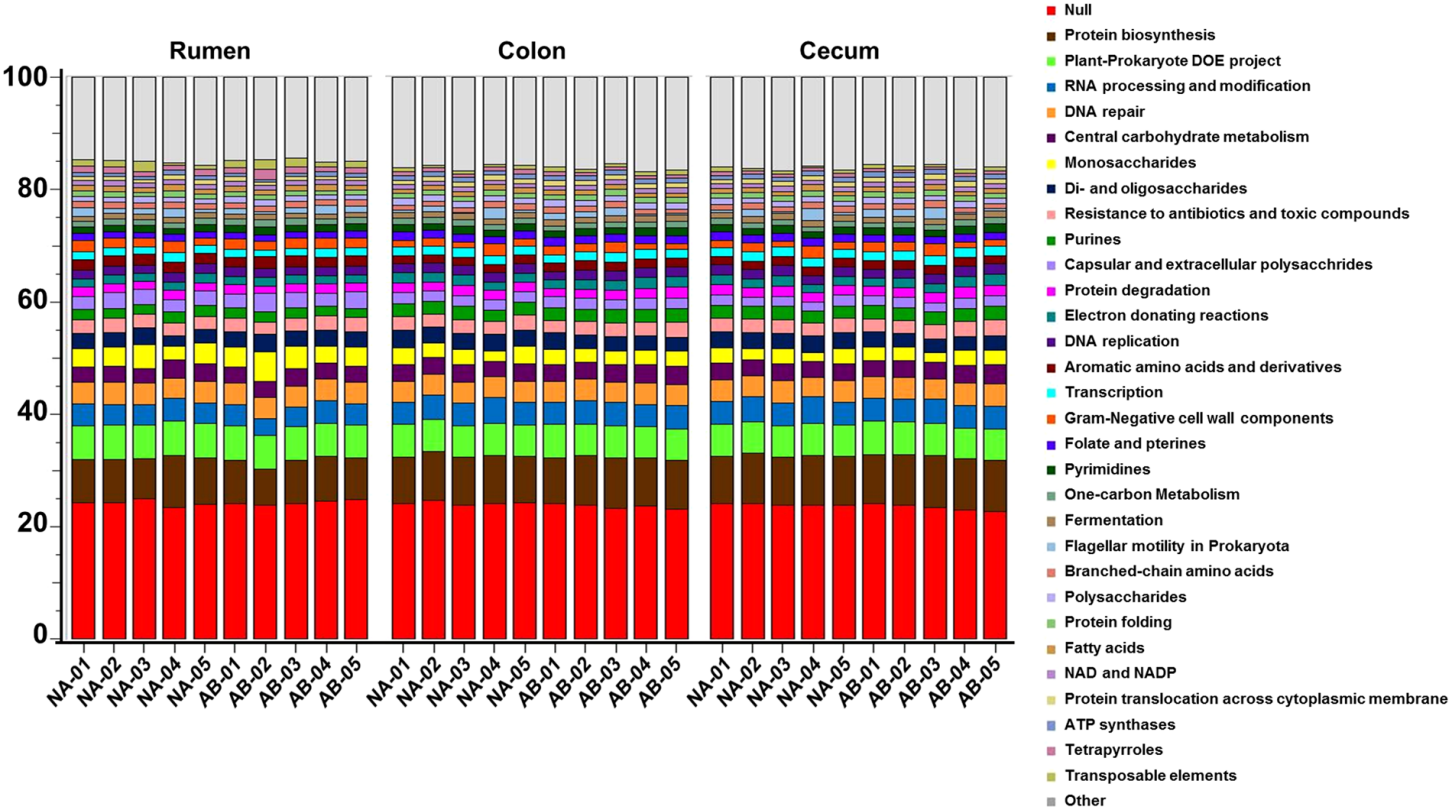
*Predicted functional profile of the microbiome in the GI tract of the steers*. The metagenomic functional analysis was performed in MG-RAST using Subsystems database with maximum e-value at 10^−5^ value, minimum percentage identity at 60%, minimum alignment length of 30 amino acids and abundance 100. Treated steers (AB) were given antibiotic feed additives and control steers (NA) were the control animals which received no treatments. The X axis denotes the samples and Y axis denotes the percentage of predominant functional genes present in the sample at level 2 hierarchy.

The analysis of enzyme functions provides a metabolic blueprint of the bacterial community. The functional changes of the microbiome in the rumen, colon, and cecum of feedlot steers fed with and without antibiotic feed additives were predicted using SEED subsystems at level 2 hierarchy in MG-RAST pipeline (Fig. 5). Overall, genes with unknown (null) functions were 24.17%, 23.81%, and 23.17% in the rumen, colon, and cecum respectively suggesting that functions of a large proportion of the genes that encodes for enzymes are yet unknown. Genes associated with protein biosynthesis were the most abundant among the known functions and the percentage was 7.91, 8.61 and 8.89 in the rumen, colon, and cecum respectively. The other dominant functions were associated with plant-prokaryote associations, RNA processing and modification, DNA repair, and central carbohydrate metabolism.

**Figure 6:**
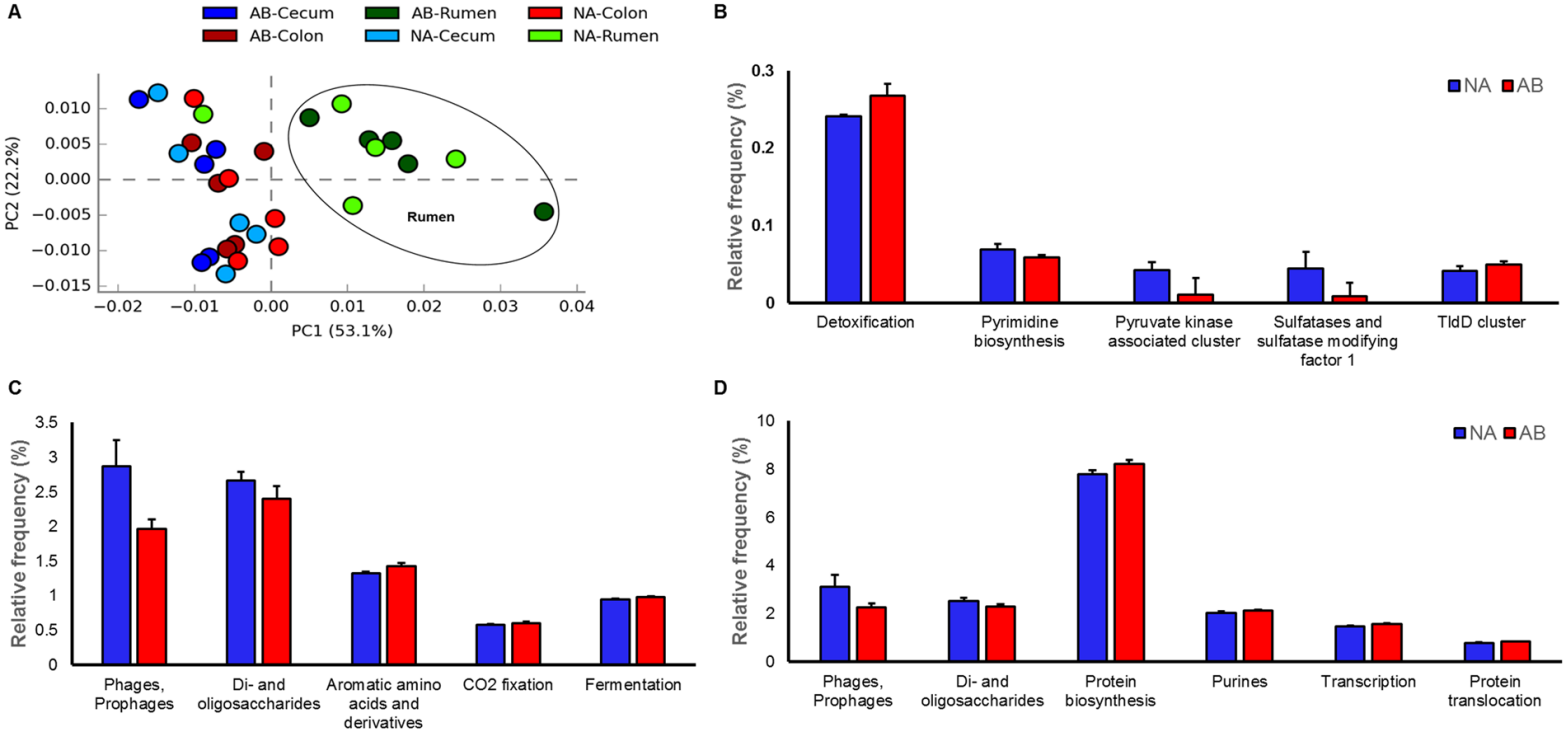
(**A**) *Principal coordinate analysis* (*PCA*) *of the functional diversity in the GI tract of steers*. Treated steers were given antibiotic feed additives and control steers were the control animals. The rumen microbiome samples, except for NA-04, clustered together and separated from cecum and colon samples. Comparison of functional genes that are significantly different (p < 0.05) between NA (blue bars) and AB steers (red bars) (**B**) in rumen (**C**) colon and D) cecum are given above.

The diversity in the functional characteristics of the metagenomes was analyzed using STAMP software. The PCA analysis of functional hierarchy showed clustering of all rumen samples, except NA-04, and segregating away from cecum and colon samples (Fig. 6A). The cecum and colon samples did not have a definite pattern of segregation and were overlapping each other. This observation for functional analysis was similar to that found for taxonomical clustering at the genera-level.

Further in-depth analysis revealed that the genes involved in detoxification were significantly increased in the rumen of AB steers (Fig. 6B). However, sulfatases and sulfatase modifying factor 1 and pyruvate kinase associated genes were decreased in this group. In colon (Fig. 6C) and cecum (Fig. 6D), the genes associated to phages and prophages as well as di- and oligo-saccharides metabolism was reduced in the AB steers.

### Resistome composition of the gut

The presence of antibiotic resistance (AMR) genes in the pathogenic bacteria has been a threat to human and animal health. Microbiome of livestock could act as a potential reservoir for AMR genes for the pathogens. Antibiotic resistome of the GIT is composed of all the AMR genes present in pathogenic and non-pathogenic bacteria. The use of antibiotics as feed additives in livestock farming has been a controversial subject because of the possibility of gut microbiome gaining AMR determinants and expanding the antibiotic resistome. Functional metagenomics provides a potential resource for detecting the existence of AMR genes and antibiotic resistome composition in the gut microbial community.

**Figure 7:**
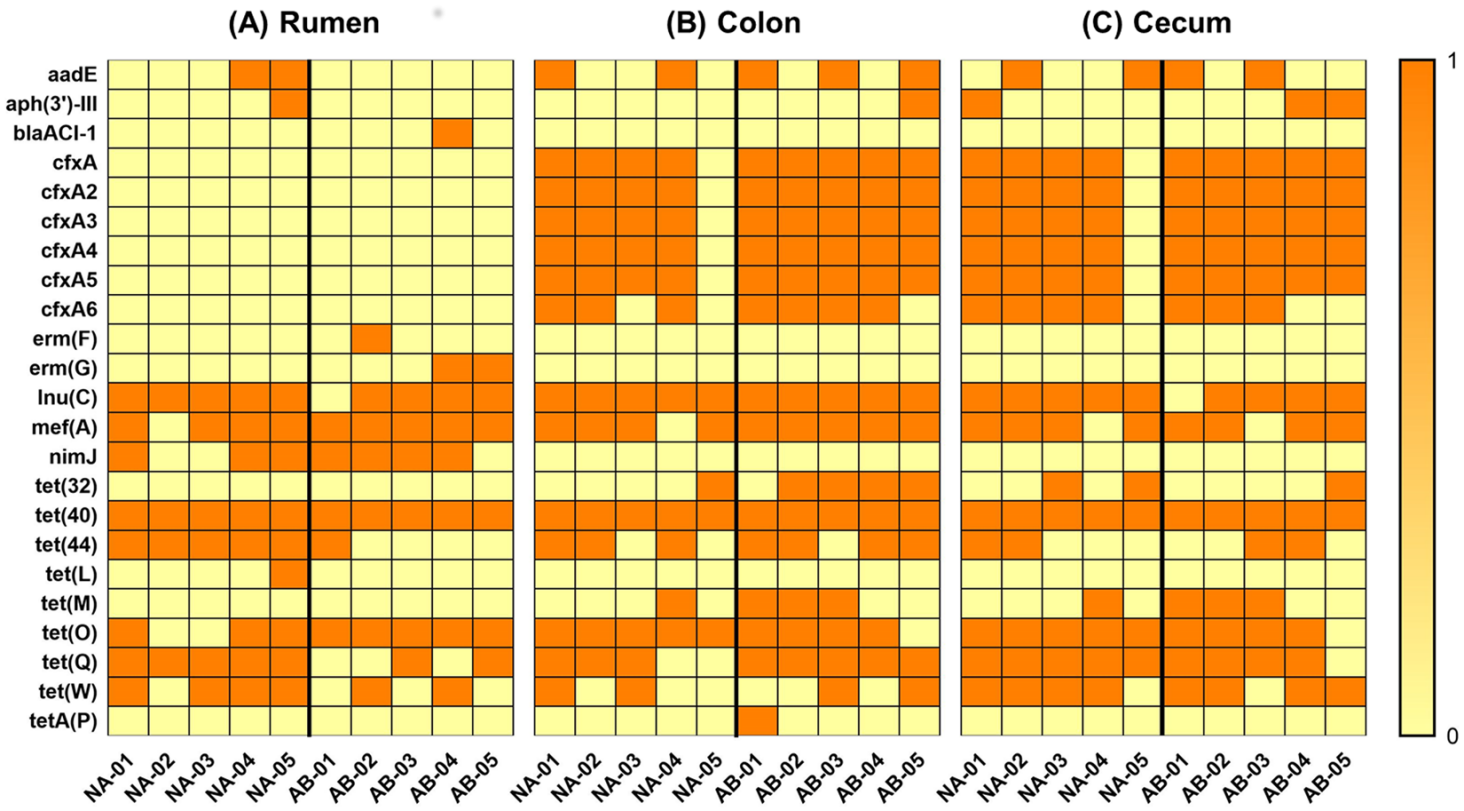
*Heatmap showing the resistome profile in the GI tract of feedlot steers*. Steers were fed Monensin and Tylosin as feed additives (AB) or control animals were not given any treatments (NA). Samples were collected from rumen, colon, and cecum and DNA was subjected to metagenome sequencing using Illumina platform. The raw files were de novo assembled and antibiotic resistance genes were identified searching the assembled contigs against a local copy of ResFinder database using BLAST. The yellow color represents absence of the antibiotic resistance genes and orange color indicates the presence. The macrolide resistance genes erm(F) and erm(G) were detected only in rumen of 3 AB steers.

In this study, the treated steers were provided tylosin and monensin. We compared the presence of AMR genes in the rumen, cecum, and colon of NA and AB steers (Fig. 7) to determine whether antibiotic feed additives could influence the presence of AMR genes and expand the antibiotic resistome. The resistance genes pattern was predicted by BLAST searching assembled metagenome contigs against the ResFinder database. The rumen microbiota of three AB steers had the presence of the macrolide resistance genes *ermF* (AB-02) and *ermG* (AB-04, AB-05). However, these genes were not detected in the rumen of NA steers or in the cecum and colon of both groups. On the contrary, aminoglycoside resistance genes *aadE* and *aph(3)-III* were present only in the rumen of two NA steers (NA-04 and NA-05) which were never administered injectable antibiotics or antibiotic feed additives. The *aadE* gene was also present in the colon samples of NA-01, NA-04, AB-01, AB-03, and AB-05 and the cecum of NA-02 and NA-05, AB-01 and AB-03. Another aminoglycoside resistance determinant *aph(3)-III* was detected in NA-01 (cecum), NA-05 (rumen), AB-04 (cecum), and AB-05 (colon and cecum). These findings suggest the wide distribution of aminoglycoside resistance determinants irrespective of the treatments in the experiment.

Furthermore, Lincosamide resistance gene (*InuC*) was found in the rumen, colon, and cecum of both control and treated groups. However, this gene was detected only in the colon of AB-01 while all other steers had it in the rumen, colon, and cecum. Different variants of tetracycline resistance genes were also ubiquitously found in the rumen, cecum, and colon of all steers, irrespective of the treatment given during the feeding trial. Interestingly, the beta-lactam resistance genes were found only in the cecum and colon of both groups of steers but not in the rumen except for AB-04 which carried *blaACl-1*. Conversely, resistance gene *nimJ* against metronidazole was observed only in the rumen of 3 NA steers and 4 AB steers but not in cecum and colon, irrespective of the treatments. The distribution of resistance genes in both group of steers points to the universal presence of antimicrobial resistance in the microbial population rather than resistance induced by treatments. Overall, no correlations were observed between the presence of AMR genes in the GIT microbiome and the administration of antibiotic feed additives.

## Discussion

This study compared the GIT microbiome and resistome of steers provided antibiotic feed additives, monensin and tylosin, with control animals that were fed the same ration but not provided antibiotics. The treated steers also received growth promoting implants and a beta-agonist during the last 31 days of the trial, both of which are common practices in conventional feedlot management^31^. Monensin, an anti-bacterial ionophore, is widely used in the feedlot industry to enhance production. It reduces the average feed intake and increases weight gain by enhancing propionic acid production in the rumen thereby resulting in improved energy efficiency^32,33^. This feed additive also selectively reduces the gram-positive bacterial population. Tylosin is also an antimicrobial agent that acts against gram-positive bacteria. The major role of tylosin is to reduce the incidence of liver abscesses when steers are fed a rapidly fermentable carbohydrate-rich diet during the finishing phase of beef production^9–11^. When fed a high concentrate diet, the rumen pH is lowered due to the production of short-chain fatty acids, particularly lactate. Reduced pH promotes proliferation of *Fusobacterium necrophorum*, the primary agent that causes liver abscesses. Tylosin apparently inhibits the growth of *F. necrophorum* in the rumen and thus reduces the liver abscess incidences^7,8^. Often monensin and tylosin are provided concurrently to cattle in North American feedlots. The active ingredient in Optaflexx is ractopamine-HCl and has been found to improve average daily gain, feed efficiency, and carcass gain^34,35^. However, there is no available information that could link Optaflexx administration and the microbial pattern in the gut. The Revalor^**®**^-200 implants, which has anabolic steroids as active ingredients, is administered to feedlot cattle to improve their growth performance and some carcass traits^36^. Recent findings suggest that both endogenous and exogenous estrogen and testosterone levels could influence the composition of gut microbiome^37–39^. If growth promoting implants could influence gut microbiome, those changes could not be discerned from that caused by antibiotic feed additives. However, we speculate that antibiotics could produce a larger and more direct impact on the microbial composition compared to anabolic steroids. This is further supported by our finding that changes in the microbial dynamics in the rumen, cecum, and colon align with the bactericidal activity of monensin and tylosin. The abundance of members belonging to gram-positive families Ruminococcaceae, Erysipelotrichaceae and Lachanospiracea were reduced in the GIT of AB steers, while members of gram-negative Veillonellaceae increased.

In the rumen, use of antibiotic feed additives was inversely associated with species diversity and richness as indicated by lower Chao1, ShannonH, and inverse Simpson indices for AB steers. Previous research also had shown that monensin decreased rumen bacterial diversity both *in vitro* and *in vivo* ^40,41^. Contrary to the findings in the rumen, the microbial population in colon and cecum samples had similar diversity for both treatment groups. This suggests that antibiotic feed additives had only a limited effect on the distal gut microbiota of steers. A primary reason could be that monensin is actively absorbed from the rumen, metabolized in the liver, and excreted through bile and only <10% of unmetabolized monensin reaches the distal gut^42^. Similar to our findings, feeding monensin to lactating Holstein cows did not change the level of gram-positive and gram-negative associated 16S rRNA gene sequences in the colon-derived library^23^.

The diversity indices and PCA profiles show that the microbial community in the rumen is different from that of distal gut irrespective of the use of antimicrobial feed additives. Studies conducted in dairy cows and a Nellore steer also found that the microbial population was significantly different between forestomach, small intestine, and large intestine^21,22^. Results from our study further corroborate their finding that GIT region is a strong determinant of microbial community structure and also that microbial population in the fecal samples may not be representative of forestomach microbiota.

The composition of the microbial population in the rumen is significantly influenced by the diet^15,16,43^. In forage fed cattle, the community is more diverse and tend to have a higher Firmicutes to Bacteroidetes ratio. Major phyla that dominate in the rumen of forage-fed animals include Firmicutes, Bacteroidetes, Fibrobacter, and Proteobacteria. As cattle transition from high forage to high concentrate diets, the diversity is lost and the composition altered^43,44^. However, changes in the microbial community due to high grain diet do not follow a definite pattern. Few studies have reported that the abundance of Firmicutes increased in the rumen of dairy cows when fed with a highly fermentable diet that induced acidosis^6,44^. On the other hand, the abundance of Bacteroidetes in the rumen increased in beef steers when 60% or more of the diet was composed of grain^16^. In the present study, > 90% of the diet was derived from corn and corn byproducts that were highly fermentable. The percentage of reads belonging to phylum Bacteroidetes in the rumen of NA and AB steers were 63.07 ± 9.19 and 68.47 ± 7.72 respectively. Furthermore, the Firmicutes constituted only 16.56 ± 3.53% and 14.04 ± 2.49% of the reads in NA and AB steers. Additionally, the Bacteroidetes predominated in both treatment groups in the cecum and colon but the percentage was lower when compared to the rumen. Our findings suggest that a high concentrate diet provides a conducive environment for the survival of the Bacteroidetes. Although there were no significant differences in the read count percentage between the two treatments, AB steers had lower Firmicutes to Bacteroidetes ratio (0.26 vs 0.20) in the rumen. This shift in the ratio is in accordance with the activity of monensin and tylosin, which inhibits the growth of gram-positive Firmicutes. The ratio observed in this study was lower than those observed in other dietary studies, which analyzed the microbial pattern in the rumen^16,44^. This could be due to the difference in the quantity of concentrate fed to the cattle in these experiments. Previous experiments used a lower proportion of concentrates (<80%) in the diet and hence the shift in microbial dynamics could be less pronounced than in the present study.

The human microbiome of a healthy adult has a higher proportion of Firmicutes than Bacteroidetes^45^. An increased Firmicutes to Bacteroidetes ratio is observed in overweight and obese people and is associated with a Western diet, which has more rapidly digestible carbohydrate content^46^. Conversely, a decrease in the ratio is correlated with weight loss^47,48^. However, these findings in human may not be applicable in ruminants because of the anatomical and physiological differences. Ruminants depend on gluconeogenesis for up to 90% of the total glucose requirements of the body^49^. Propionic acid, a major precursor for glucose, is actively absorbed from the rumen and is converted to glucose in the liver. Phylum Bacteroidetes, in general, produces acetate and propionate as end-products of fermentation^50^. Therefore, a higher proportion of Bacteroidetes in the rumen could be favorable for efficient feed utilization and body weight gain.

At the genera-level, Prevotella was the most common genus in the rumen and second-most abundant genus in cecum and colon. This was in accordance with several studies which described Prevotella as the most abundant Bacteroidetes member found in rumen^5,15,16,22,25,51^. It is also one of the core members found in rumen microbial population^5,51^ irrespective of diet and location. Furthermore, the proportion of Prevotella in the rumen could increase up to 10 times in grain-fed cattle when compared to forage-fed animals^5,16,18,52^. Prevotella could utilize lactic acid and convert it into propionic acid. Thus, an increase in abundance of Prevotella is beneficial for high concentrate-fed cattle. Additionally, it was found that the abundance of Megasphaera and Selenomonas populations also increase when cattle are gradually adapted to a high concentrate diet^16,18,44^. Megasphaera and Selenomonas are members of Veilonellacea family and are propionic acid producers. In this study, the use of antimicrobial feed additives further increased the abundance of Megasphaera, Selenomonas, Mitsuokella, and Veilonella in the rumen. Even in cecum and colon, unclassified members of Erysipelotrichaceae and Lachnospiraceae were reduced and partially replaced by members of Veillonellaceae family. This shift in microbial population could prevent the accumulation of lactic acid and also increase the propionic acid synthesis.

Another core rumen microbial population is the genus Ruminococcus, which is composed of cellulolytic and lactic acid producers^5^. Previous research has shown that the abundance of Ruminococcus decreased when animals were transferred from a forage-based diet to a high grain diet^5,6,18^. We observed that monensin and tylosin further reduced the abundance of Ruminococcus genus in the rumen. This will be beneficial for cattle that are maintained on a high concentrate diet because of the decreased lactic acid production and associated risk of acidosis. However, the reduction in abundance of genera Ruminococcus, Erysipelotrichaceae and Lachanospira in various compartments of AB steers may impact the ability of GIT pathogen exclusion capacity of these animals. Various studies in humans have reported that these genera are important in suppressing the growth of enteric pathogens in the gut^3,53^.

We also characterized the gene functions of the bacteria using level 2 Hierarchical classification in MG-RAST. Our findings indicated that there are functional differences between the GIT regions. Similar variability in gene functions was observed in the GIT of the dairy cattle^22^. The most abundant gene functions were related to protein synthesis, RNA processing, DNA repair, carbohydrate metabolism, and resistance to antimicrobials and toxic compounds. These functions are essential for the survival and proliferation of the microbial community. When the antibiotic feed additives were included in the diet, steers had a higher abundance of genes associated with detoxification in the rumen. However, genes associated with detoxification formed less than 0.3% of the total functional genes and could be of lesser impact. In cecum and colon, the use of monensin and tylosin is correlated with reduced copy of genes for di- and oligosaccharide metabolism and genes associated with phage. One limitation of this study is the relatively smaller sample size (n = 5) used. In the present study, animals were managed under similar conditions and the data obtained, albeit small sample size, is relevant. Also, the higher cost associated with shotgun metagenome sequencing restricts using a larger sample size. A study conducted in dairy cows to analyze the microbiome changes associated with bovine dermatitis, used 4 diseased animals and 8 healthy control animals^54^. Another study that determined the rumen microbial changes associated with wheat induced frothy bloat used a total of 6 steers^25^. Similarly, 3 dairy cows were used to assess the microbial dynamics associated with lactation^24^.

In this study, we also characterized the antimicrobial resistance (AMR) genes present in the GIT of the feedlot animals. The AMR genes were identified using a local copy of ResFinder database in CLC workbench 9.4 and the parameters used were ≥85% amino acid identity and ≥50% sequence length. The whole genome sequencing of non-typhoidal *Salmonella* ^55^, *Camppylobacter* ^56^, and *Escherichia coli* ^57^ using these parameters gave a correlation between phenotypic and genotypic anti-microbial activity approaching 100%. This suggests that AMR prediction using genome sequencing is accurate and could be used as a surveillance tool. However, there was discrepancy reported in the prediction of resistance towards aminoglycoside and beta-lactams in these studies and the highest incongruity was observed for streptomycin.

Recent experiments have used shotgun metagenome sequencing as a diagnostic tool to predict AMR in swine^58^, environment^59,60^, bovine^54,61^, and human^62^ associated microbiome. Environmental samples collected from beef pens and abattoir revealed AMR determinants against 17 classes of drugs^59^. Similarly, fecal samples obtained from 6 beef heifers possessed AMR determinants against multiple drugs^61^. This study would be the first to report AMR determinants in the GIT of feedlot animals. There were AMR determinants against 8 classes of drugs in the various compartments. One of the major findings was that use of tylosin did not increase the presence of AMR genes in the AB steers. Tylosin resistance genes *tlr, ermB*, and *ermX* were not detected in any of the samples. Although macrolide resistance genes *ermF* and *ermG* were present only in the rumen of AB steers, the gene encoding for macrolide efflux pump, *mefA*, was ubiquitously found in all three compartments of both treatment groups. This is in contrast to the earlier finding where the use of tylosin increased the presence of macrolide resistance genes in feedlot steers^63,64^. Another finding was that the distribution of *cfxA* and *nimJ* genes, which provide resistance to beta-lactams and metronidazole respectively, differed between forestomach and hind-gut. This could indicate that sampling feces alone might not reveal the whole picture on AMR in the case of ruminants. Sampling forestomach along with fecal samples would be more effective in predicting a comprehensive AMR profile although collecting rumen samples would be difficult in farm conditions. Overall, our results did not show significant difference in AMR profile of steers in NA and AB groups. Since the total duration of antibiotic feed additive in this study was only 74 days, the AMR profile we discovered here may not be representation of longer term effect of such feed additives.

## Materials and Methods

### Ethics Statement

All procedures and protocols used in this study were reviewed and approved by the Institutional Animal Care and Use Committee (IACUC) at the South Dakota State University, Brookings, SD.

### Animal experiments

Simmental x Angus crossbred steers raised from a common herd were stratified to treatment according to dam age, calf birth date, and weight. Treatments included non-antibiotic control cattle (NA) and implanted cattle fed a beta-agonist and antibiotic feed additives (AB). A subset of 5 steers per treatment was randomly selected for digesta collection based upon plant entry order. Steers selected for analysis from both treatment groups were confirmed to never have received injectable antibiotics. During production, cattle were housed and managed according to the approved Institutional Animal Care and Use Committee protocol.Prior to weaning, steer calves were managed at the SDSU Antelope Field Station near Buffalo, S.D. where they were randomly designated to treatment after being stratified by cow age, calf birth date, and weight. Steers within the AB group received calf hood implants (36 mg zeranol; Ralgro, Merck Animal Health) at an average 74 days of age. Implants were administered subcutaneously in the middle one-third of the ear of the AB group only. After weaning, all steers received the same diet during morning feed delivery. Steers were backgrounded for a total of 71 days on a high roughage ration (grass hay, concentrate pellets, dry corn cobs, glycerin, distillers grains, limestone, and minerals). During backgrounding, AB steers were re-implanted (80 mg trenbolone acetate and 16 mg estradiol; Revalor^**®**^-IS, Merck Animal Health) at an average of 235 days of age.

### Feedlot Management

To be finished, steers were maintained within assigned feedlot pens and acclimated to the finishing diet using five step-up diets over a period of 68 days. The final finishing diet was composed of 40% wet corn gluten feed (Sweet Bran, Cargill Inc., Blair, NE), 48% dry-rolled corn, 7% grass hay, and 5% supplement containing 58.25% ground corn, 29.57% limestone, 5.59% iodized salt, 4.65% ammonium chloride, 0.93% trace mineral mix, 0.25% thiamine, and 0.21% Vitamins A, D, and E. Nutrient composition was 72.58% DM, 13.9% CP, 2.04 Mcal/kg NE_m_, and 1.38 Mcal/kg NE_g_. Steers in the AB group received 478.3 g/ton monensin (Monensin 90, Elanco Animal Health, Greenfield, IN) and 96.1 g/ton tylosin (Tylosin 40, Elanco Animal Health) as a pre-mixed supplement from the beginning of the finishing period to harvest (approximately 5 months). Whereas, the steers in the NA group never received hormone implants, ionophores, beta-agonists, feed-grade or injectable antibiotics or antimicrobials.

During the finishing period steers within AB group received a final implant (200 mg trenbolone acetate and 20 mg estradiol; Revalor^**®**^-200, Merck Animal Health) at an average of 330 days of age. Additionally, steers in the AB group received a beta-agonist (ractopamine-HCI; Optaflexx^®^-45, Elanco Animal Health) during the final 31 days prior to finishing. At harvest, which was based on a target back fat thickness of 1.5 cm, the average age of NA steers were 13 months and AB steers were 14 months. The average dry matter intake (DMI) was greater for AB (12.58 kg ± 0.23) than NA (11.54 kg ± 0.24; *P* < 0.0001). Average daily gains (ADG) were also greater for AB (1.79 kg ± 0.05) than NA (1.54 ± 0.05; *P* < 0.0001). All cattle had ad libitum access to fresh water throughout the study.

### Sample collection and genomic DNA isolation

Samples of digesta were collected from rumen, colon, and cecum immediately after slaughter and were snap-frozen in liquid nitrogen. These samples were then transferred to −80 °C and stored until use. Genomic DNA was isolated from these samples using Powersoil DNA isolation kit (Mo Bio Laboratories Inc, CA). Approximately 100 mg of sample contents were transferred to bead tubes for DNA extraction. After adding 60 µl of solution C1 to the bead tubes, samples were homogenized for 2 min using Tissuelyser (Qiagen, MD). Remaining steps of DNA isolation was performed according to the manufacturer’s protocol. The DNA was eluted in 50 µL nuclease free water. The quality of isolated DNA was analyzed using NanoDrop™ One (Thermo Scientific™, DE) and DNA was stored at −20 °C until use.

### Microbial DNA enrichment and sequencing

Selective enrichment of microbial genomic DNA was performed using NEBNext® Microbiome DNA Enrichment Kit (New England Biolabs, Inc. MA)^26^. The enrichment process was performed according to manufacturer’s protocol. Briefly, 0.5 µg of genomic DNA was treated with 80 µl of MBD2-Fcbound magnetic beads in the presence of binding buffer and incubated at room temperature for 15 min with rotation. After incubation, beads were separated by keeping the tubes on a magnetic rack for 5 min. The supernatant, which contained microbial DNA, was transferred to another tube and were purified using Agencourt AMPure XP beads (Beckman Coulter) and stored at −20 °C.

Enriched microbial genomic DNA from test samples was used for shotgun metagenome sequencing. The concentrations of genomic DNA samples were measured using Qubit Fluorometer 3.0 (Invitrogen, Carlsbad, CA) and the concentration was adjusted to 0.2 ng/µl. After normalization, sequencing libraries were prepared using Nextera XT DNA Sample Prep Kit (Illumina Inc. San Diego, CA). Tagmentation of samples using 1 ng of template was conducted according to manufacturer’s protocol, followed by PCR amplification using a unique combination of barcode primers. PCR products were then cleaned up using Agencourt AMpure XP beads (Beckman Coulter). Purified products were normalized using library normalization protocol suggested by manufacturer. Equal volumes of normalized libraries were pooled together and diluted in hybridization buffer. The pooled libraries were heat denatured and spiked with 5% of the Illumina PhiX control DNA prior to loading the sequencer. Illumina paired-end sequencing was performed on the Miseq platform using a 2 × 250 paired-end sequencing chemistry.

### Sequence data processing

The raw data files were de-multiplexed and converted to FASTQ files using Casava v.1.8.2. (Illumina, Inc, San Diego, CA, USA). The raw sequence data for all the samples in this study has been deposited in NCBI Sequence Read Archive (NCBI SRA) under the bioproject number - PRJNA390551. FASTQ files were concatenated and analyzed using MG-RAST^65^. The sequences were subjected to quality control in MG-RAST. This included de-replication, removal of host specific sequences, ambiguous base filtering, and length filtering. The taxonomical abundance of organisms was analyzed using MG-RAST with Best Hit Classification approach using Refseq database and parameters were limited to minimum e-value of 10^−5^, minimum percentage identity of 60%, a minimum abundance of 100, and a minimum alignment length of 30 amino acids. The functional abundance was analyzed using Hierarchical Classification in MG-RAST using Subsystems and parameters were limited to minimum e-value of 10^−5^, minimum percentage identity of 60%, a minimum abundance of 100, and a minimum alignment length of 30. The OTU abundance tables were downloaded from MG-RAST and were used for downstream statistical analysis.

To determine the resistome composition of the samples, raw data files were assembled in CLC workbench 9.4 using *de novo* metagenome assembler (Qiagen Bioinformatics, CA). The assembled metagenome was searched against a local copy of ResFinder database^66^ using BLAST.

### Statistical analysis

Influence of AB and NA on animal performance (DMI and ADG) were evaluated using PROC MIXED in SAS. Cow age was used as a covariate and LS-means were computed for response variables. The significance level was set at P < 0.05. The phyla distribution was statistically analyzed using ANOVA with Tukey’s multiple comparison test. Indices of taxonomical diversity in the samples was determined using Explicet software^67^. Bootstrapping was performed to reduce the biases in sequencing depth. A total of 50 bootstraps were conducted to calculate the α-diversity indices –Shannon, Simpson, and Chao1. Diversity between samples (β-diversity) was estimated by deriving Bray-Curtis index. Statistical differences among Firmicutes and Bacteroidetes in various digestive tract segments were calculated using STAMP software^68^. The differences were estimated using two-sided Welch’s t-test. Similar parameters were used for analyzing the statistical differences among overall functional abundance. Corrected p-values below 0.05 were considered significant. The principal component analysis (PCA) of gene functions and taxonomical distribution were also generated using STAMP software.

## References

[1] Clarke TB (2010). Recognition of peptidoglycan from the microbiota by Nod1 enhances systemic innate immunity. Nat Med.

[2] Flint HJ, Scott KP, Louis P, Duncan SH (2012). The role of the gut microbiota in nutrition and health. Nat Rev Gastroenterol Hepatol.

[3] Buffie CG, Pamer EG (2013). Microbiota-mediated colonization resistance against intestinal pathogens. Nat Rev Immunol.

[4] Scott KP, Gratz SW, Sheridan PO, Flint HJ, Duncan SH (2013). The influence of diet on the gut microbiota. Pharmacol Res.

[5] Henderson G (2015). Rumen microbial community composition varies with diet and host, but a core microbiome is found across a wide geographical range. Sci Rep.

[6] Tajima K (2000). Rumen Bacterial Community Transition During Adaptation to High-grain Diet. Anaerobe.

[7] Nagaraja TG, Chengappa MM (1998). Liver abscesses in feedlot cattle: a review. J Anim Sci.

[8] Nagaraja TG, Sun Y, Wallace N, Kemp KE, Parrott CJ (1999). Effects of tylosin on concentrations of Fusobacterium necrophorum and fermentation products in the rumen of cattle fed a high-concentrate diet. Am J Vet Res.

[9] Meyer NF (2009). Effect of essential oils, tylosin, and monensin on finishing steer performance, carcass characteristics, liver abscesses, ruminal fermentation, and digestibility. J Anim Sci.

[10] Potter EL (1985). Effect of monensin and tylosin on average daily gain, feed efficiency and liver abscess incidence in feedlot cattle. J Anim Sci.

[11] Stock RA (1995). Effect of monensin and monensin and tylosin combination on feed intake variation of feedlot steers. J Anim Sci.

[12] Aowicki D, Huczynski A (2013). Structure and antimicrobial properties of monensin A and its derivatives: summary of the achievements. Biomed Res Int.

[13] Knothe H (1977). A review of the medical considerations of the use of tylosin and other macrolide antibiotics as additives in animal feeds. Infection.

[14] Wright GD (2007). The antibiotic resistome: the nexus of chemical and genetic diversity. Nat Rev Microbiol.

[15] de Menezes AB (2011). Microbiome analysis of dairy cows fed pasture or total mixed ration diets. FEMS Microbiol Ecol.

[16] Fernando SC (2010). Rumen microbial population dynamics during adaptation to a high-grain diet. Appl Environ Microbiol.

[17] Goad DW, Goad CL, Nagaraja TG (1998). Ruminal microbial and fermentative changes associated with experimentally induced subacute acidosis in steers. J Anim Sci.

[18] Tajima K (2001). Diet-Dependent Shifts in the Bacterial Population of the Rumen Revealed with Real-Time PCR. Applied and Environmental Microbiology.

[19] Weimer PJ, Stevenson DM, Mertens DR, Thomas EE (2008). Effect of monensin feeding and withdrawal on populations of individual bacterial species in the rumen of lactating dairy cows fed high-starch rations. Appl Microbiol Biotechnol.

[20] De Nardi R (2016). Metagenomic analysis of rumen microbial population in dairy heifers fed a high grain diet supplemented with dicarboxylic acids or polyphenols. BMC Vet Res.

[21] de Oliveira MNV (2013). Characterizing the microbiota across the gastrointestinal tract of a Brazilian Nelore steer. Veterinary Microbiology.

[22] Mao S, Zhang M, Liu J, Zhu W (2015). Characterising the bacterial microbiota across the gastrointestinal tracts of dairy cattle: membership and potential function. Sci Rep.

[23] McGarvey JA, Hamilton SW, DePeters EJ, Mitloehner FM (2010). Effect of dietary monensin on the bacterial population structure of dairy cattle colonic contents. Appl Microbiol Biotechnol.

[24] Pitta DW (2016). Metagenomic assessment of the functional potential of the rumen microbiome in Holstein dairy cows. Anaerobe.

[25] Pitta DW (2016). Metagenomic Analysis of the Rumen Microbiome of Steers with Wheat-Induced Frothy Bloat. Front Microbiol.

[26] Feehery GR (2013). A method for selectively enriching microbial DNA from contaminating vertebrate host DNA. PLoS One.

[27] Willing BP, Russell SL, Finlay BB (2011). Shifting the balance: antibiotic effects on host-microbiota mutualism. Nat Rev Microbiol.

[28] Ferrer M, M dos Santos VA, Ott SJ, Moya A (2014). Gut microbiota disturbance during antibiotic therapy: a multi-omic approach. Gut Microbes.

[29] Suchodolski JS (2009). The effect of the macrolide antibiotic tylosin on microbial diversity in the canine small intestine as demonstrated by massive parallel 16S rRNA gene sequencing. BMC Microbiol.

[30] Kim M, Eastridge ML, Yu Z (2014). Investigation of ruminal bacterial diversity in dairy cattle fed supplementary monensin alone and in combination with fat, using pyrosequencing analysis. Can J Microbiol.

[31] Garmyn AJ, Miller MF (2014). MEAT SCIENCE AND MUSCLE BIOLOGY SYMPOSIUM–implant and beta agonist impacts on beef palatability. J Anim Sci.

[32] Ipharraguerre IR, Clark JH (2003). Usefulness of ionophores for lactating dairy cows: a review. Animal Feed Science and Technology.

[33] Ellis JL (2012). Quantifying the effect of monensin dose on the rumen volatile fatty acid profile in high-grain-fed beef cattle. J Anim Sci.

[34] Bittner CJ (2016). Effect of ractopamine hydrochloride (Optaflexx) dose and duration on growth performance and carcass characteristics of finishing steers. J Anim Sci.

[35] Quinn MJ (2008). The effects of ractopamine-hydrogen chloride (Optaflexx) on performance, carcass characteristics, and meat quality of finishing feedlot heifers. J Anim Sci.

[36] Duckett SK, Pratt SL (2014). MEAT SCIENCE AND MUSCLE BIOLOGY SYMPOSIUM–anabolic implants and meat quality. J Anim Sci.

[37] Javurek AB (2016). Effects of exposure to bisphenol A and ethinyl estradiol on the gut microbiota of parents and their offspring in a rodent model. Gut Microbes.

[38] Flores R (2012). Fecal microbial determinants of fecal and systemic estrogens and estrogen metabolites: a cross-sectional study. J Transl Med.

[39] Harada N (2016). Castration influences intestinal microflora and induces abdominal obesity in high-fat diet-fed mice. Sci Rep.

[40] Scharen M (2017). Differential effects of monensin and a blend of essential oils on rumen microbiota composition of transition dairy cows. J Dairy Sci.

[41] Johnson MC, Devine AA, Ellis JC, Grunden AM, Fellner V (2009). Effects of antibiotics and oil on microbial profiles and fermentation in mixed cultures of ruminal microorganisms. J Dairy Sci.

[42] Herberg R, Manthey J, Richardson L, Cooley C, Donoho A (1978). Excretion and tissue distribution of [14C]monensin in cattle. Journal of Agricultural and Food Chemistry.

[43] Belanche A (2012). Shifts in the rumen microbiota due to the type of carbohydrate and level of protein ingested by dairy cattle are associated with changes in rumen fermentation. J Nutr.

[44] Khafipour E, Li S, Plaizier JC, Krause DO (2009). Rumen microbiome composition determined using two nutritional models of subacute ruminal acidosis. Appl Environ Microbiol.

[45] Mariat D (2009). The Firmicutes/Bacteroidetes ratio of the human microbiota changes with age. BMC Microbiol.

[46] Chakraborti CK (2015). New-found link between microbiota and obesity. World J Gastrointest Pathophysiol.

[47] De Filippo C (2010). Impact of diet in shaping gut microbiota revealed by a comparative study in children from Europe and rural Africa. Proc Natl Acad Sci USA.

[48] Ley RE, Turnbaugh PJ, Klein S, Gordon JI (2006). Microbial ecology: human gut microbes associated with obesity. Nature.

[49] Young JW (1977). Gluconeogenesis in cattle: significance and methodology. J Dairy Sci.

[50] Macfarlane S, Macfarlane GT (2003). Regulation of short-chain fatty acid production. Proc Nutr Soc.

[51] Jami E, Mizrahi I (2012). Composition and similarity of bovine rumen microbiota across individual animals. PLoS One.

[52] Shanks OC (2011). Community structures of fecal bacteria in cattle from different animal feeding operations. Appl Environ Microbiol.

[53] Pamer EG (2016). Resurrecting the intestinal microbiota to combat antibiotic-resistant pathogens. Science.

[54] Zinicola M (2015). Shotgun Metagenomic Sequencing Reveals Functional Genes and Microbiome Associated with Bovine Digital Dermatitis. PLOS ONE.

[55] McDermott PF (2016). Whole-Genome Sequencing for Detecting Antimicrobial Resistance in Nontyphoidal Salmonella. Antimicrob Agents Chemother.

[56] Zhao S (2015). Whole-Genome Sequencing Analysis Accurately Predicts Antimicrobial Resistance Phenotypes in Campylobacter spp. Appl Environ Microbiol.

[57] Tyson GH (2015). WGS accurately predicts antimicrobial resistance in Escherichia coli. J Antimicrob Chemother.

[58] Munk P (2017). A sampling and metagenomic sequencing-based methodology for monitoring antimicrobial resistance in swine herds. J Antimicrob Chemother.

[59] Noyes NR (2016). Resistome diversity in cattle and the environment decreases during beef production. Elife.

[60] Bengtsson-Palme J, Boulund F, Fick J, Kristiansson E, Larsson DG (2014). Shotgun metagenomics reveals a wide array of antibiotic resistance genes and mobile elements in a polluted lake in India. Front Microbiol.

[61] Durso LM, Harhay GP, Bono JL, Smith TPL (2011). Virulence-associated and antibiotic resistance genes of microbial populations in cattle feces analyzed using a metagenomic approach. Journal of Microbiological Methods.

[62] Andersen H (2016). Use of Shotgun Metagenome Sequencing To Detect Fecal Colonization with Multidrug-Resistant Bacteria in Children. J Clin Microbiol.

[63] Beukers AG (2015). Effect of in-feed administration and withdrawal of tylosin phosphate on antibiotic resistance in enterococci isolated from feedlot steers. Front Microbiol.

[64] Jacob ME (2008). Effects of feeding wet corn distillers grains with solubles with or without monensin and tylosin on the prevalence and antimicrobial susceptibilities of fecal foodborne pathogenic and commensal bacteria in feedlot cattle. J Anim Sci.

[65] Meyer F (2008). The metagenomics RAST server - a public resource for the automatic phylogenetic and functional analysis of metagenomes. BMC Bioinformatics.

[66] Zankari E (2012). Identification of acquired antimicrobial resistance genes. J Antimicrob Chemother.

[67] Robertson CE (2013). Explicet: graphical user interface software for metadata-driven management, analysis and visualization of microbiome data. Bioinformatics.

[68] Parks DH, Tyson GW, Hugenholtz P, Beiko RG (2014). STAMP: statistical analysis of taxonomic and functional profiles. Bioinformatics.

